# Nanoengineered Gallium Ion Incorporated Formulation for Safe and Efficient Reversal of PARP Inhibition and Platinum Resistance in Ovarian Cancer

**DOI:** 10.34133/research.0070

**Published:** 2023-03-09

**Authors:** Yangyang Li, Yixuan Cen, Mengyan Tu, Zhenzhen Xiang, Sangsang Tang, Weiguo Lu, Hongbo Zhang, Junfen Xu

**Affiliations:** ^1^ Zhejiang Provincial Key Laboratory of Precision Diagnosis and Therapy for Major Gynecological Diseases, Women’s Hospital, Zhejiang University School of Medicine, Hangzhou, Zhejiang, China.; ^2^ Department of Gynecologic Oncology, Women’s Hospital, Zhejiang University School of Medicine, Hangzhou 310006, Zhejiang, China.; ^3^ Women’s Reproductive Health Laboratory of Zhejiang Province, Women’s Hospital, Zhejiang University School of Medicine, Hangzhou 310006, Zhejiang, China.; ^4^ Cancer Center, Zhejiang University, Hangzhou 310058, Zhejiang, China.; ^5^ Pharmaceutical Sciences Laboratory, Åbo Akademi University, Turku FI-20520, Finland.; ^6^ Turku Bioscience Centre, University of Turku and Åbo Akademi University, Turku FI-20520, Finland.

## Abstract

Platinum-based chemotherapy remains the main systemic treatment of ovarian cancer (OC). However, the inevitable development of platinum and poly (adenosine diphosphate-ribose) polymerase inhibitor (PARPi) resistance is associated with poor outcomes, which becomes a major obstacle in the management of this disease. The present study developed “all-in-one” nanoparticles that contained the PARPi olaparib and gallium (Ga) (III) (olaparib-Ga) to effectively reverse PARPi resistance in platinum-resistant A2780-cis and SKOV3-cis OC cells and in SKOV3-cis tumor models. Notably, the olaparib-Ga suppressed SKOV3-cis tumor growth with negligible toxicity. Moreover, the suppression effect was more evident when combining olaparib-Ga with cisplatin or carboplatin, as evaluated in A2780-cis and SKOV3-cis cells. Mechanistically, the combined treatment induced DNA damage, which elicited the activation of ataxia telangiectasia mutated (ATM)/AMT- and Rad3-related (ATR) checkpoint kinase 1 (Chk1)/Chk2 signal transduction pathways. This led to the arrest of cell cycle progression at S and G_2_/M phases, which eventually resulted in apoptosis and cell death due to unrepairable DNA damage. In addition, effective therapeutic responses to olaparib-Ga and cisplatin combination or olaparib-Ga and carboplatin combination were observed in SKOV3-cis tumor-bearing animal models. Altogether, the present findings demonstrate that olaparib-Ga has therapeutic implications in platinum-resistant OC cells, and the combination of olaparib-Ga with cisplatin or carboplatin may be promising for treating patients with OC who exhibit resistance to both PARPi and platinum.

## Introduction

Ovarian cancer (OC) is the main cause of mortality because of female reproductive cancer [1,2]. Despite extensive investigation to understand the mechanisms of OC, standard therapeutic treatments are still based on optimal cyto-reductive surgery and platinum-based chemotherapy. However, the majority of patients succumb to OC recurrence, because >80% of cases become treatment resistant, with > 207,000 mortalities yearly worldwide, resulting in a 30% 5-year survival rate [3]. Targeted strategies are widely used in cancer treatment. The Food and Drug Administration has approved the use of poly (adenosine diphosphate (ADP)-ribose) polymerase inhibitors (PARPi) for patients with OC [4]. However, almost invariably, the tumors eventually develop resistance to chemotherapy and PARPi, thus limiting the efficacy of OC treatment. Therefore, developing efficacious therapies for resistant OC remains an active area of research, which will be of important benefit to the survival of patients.

Platinum-based compounds, particularly cisplatin and carboplatin, exert anticancer activity prominently through causing DNA damage, followed by activating the DNA damage response and promoting apoptosis [5]. Although tumors have an initial sensitivity to platinum-base therapies, they often eventually develop chemoresistance in patients with OC. It has been found that resistance to platinum may depend on the upregulation of the transporter gene adenosine triphosphate binding cassette subfamily B member 1 [6], reversion of breast cancer gene (*BRCA1*) and *BRCA2* germ line alleles [7–9] or impaired apoptotic pathways [5,10]. These considerations also apply for PARPi resistance. Platinum resistance and tumor homologous recombination (HR) proficiency can explain PARPi resistance [11,12]. Increased drug efflux [13,14], loss of PARPi function [15,16], reactivation of HR [6,17], loss of poly (ADP-ribose) glycohydrolase [18,19], and stabilization of the replication fork [20,21] can contribute to a PARPi-resistant phenotype. Thus, there is an urgent need to develop reliable treatments to overcome platinum and PARPi drug resistance.

The fabrication of metal nanoparticles has recently received intense attention for the treatment of cancers including OC [22–27]. For instance, it has been reported that iron (Fe) oxide nanoparticles could exert remarkable anticancer activity against OC cells [28,29]. Gallium (Ga) is second after platinum in its use in cancer treatment [30,31]. Ga-based nanoparticles have demonstrated anticancer activities in malignancies, such as lung, prostate, and breast cancer [32–34]. The main mechanisms of Ga in cancer therapy have been ascribed to the resemblance of Ga^3+^ to Fe^3+^, resulting in Fe deprivation and subsequent inhibited activity of several enzymes and mitochondrial-dependent apoptosis [35–39]. Our group has recently combined the PARPi olaparib with Ga (III) nanoparticles (olaparib-Ga) to effectively retrain HR-proficient OC cell proliferation and tumor growth [40]. However, the effects and mechanisms of olaparib-Ga nanodrug on resistant OC cells remain unknown.

It was hypothesized that olaparib-Ga could also increase the DNA damage and tumor cell death in resistant OC cells. The present study demonstrated that olaparib-Ga resensitized platinum-resistant A2780-cis and SKOV3-cis OC cells to PARPi, resulting in increased DNA damage, apoptosis, and tumor cell death. This nanodrug exhibited enhanced antitumor efficiency against SKOV3-cis cells in vivo. Notably, olaparib-Ga could be used in therapeutic strategies for resistant OC cells. The current study revealed that olaparib-Ga in combination with cisplatin or carboplatin effectively recovered the sensitivity to platinum in resistant OC cells. The present study better characterized the understanding of the mechanism of the drug combination to improve antitumor response. The current results showed that the combinations could induce DNA damage, followed by the activation of ataxia telangiectasia mutated (ATM) and AMT- and Rad3-related (ATR), which subsequently activated the checkpoint kinase 1 (Chk1)/Chk2 cell cycle checkpoints. The activities of cyclin A/cyclin-dependent kinase 2 (CDK2) and cyclin B1/CDK1 were then suppressed by the activation of the cell cycle checkpoints, and cell cycle progression was arrested at the S and G_2_/M phases. Cells with irreparable DNA damage eventually underwent apoptosis and cell death. The present study also showed that combined therapy significantly suppressed tumor growth in the SKOV3-cis tumor-bearing animal models. These novel findings suggest that olaparib-Ga represents a promising nanodrug to overcome PARPi and platinum resistance in OC, which is of great importance for improving the therapeutic outcomes of this disease.

## Results

### Characterization of olaparib-Ga in platinum-resistant OC cells

**Fig. 1.**
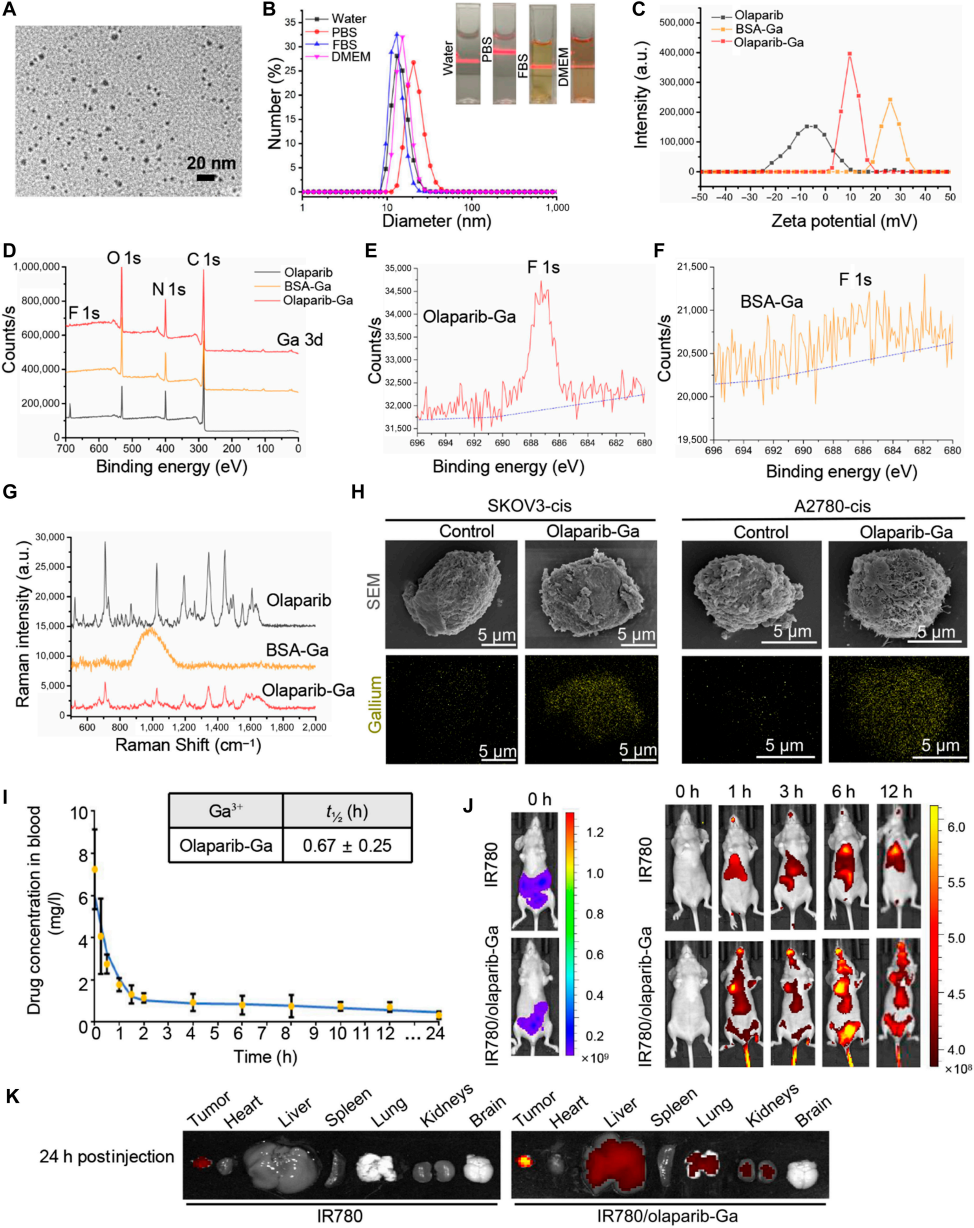
Characterization of olaparib-Ga nanodrug. (A) Representative transmission electron microscopy micrographs of olaparib-Ga nanoparticles. Scale bar, 20 nm. (B) Dynamic light scattering data of olaparib-Ga in different solutions. The inserted pictures showed the evaluation of the stability of olaparib-Ga in various solutions. (C) Zeta potential of olaparib, BSA-Ga, and olaparib-Ga. (D) X-ray photoelectron spectroscopy was used to determine the spectra of olaparib, BSA-Ga, and olaparib-Ga. (E and F) F 1s electronic energy of olaparib-Ga (E) and BSA-Ga (F). (G) Raman shifts of olaparib, BSA-Ga, and olaparib-Ga excited by a 700-nm laser. (H) The presence of Ga^3+^ in the SKOV3-cis and A2780-cis cells was confirmed by scanning electron microscopy and element mapping. (I) Healthy mice received an intravenous injection of olaparib-Ga. The concentration of Ga^3+^ at different time points (0, 0.25, 0.5, 1, 1.5, 2, 4, 6, 8, 10, 12, and 24 h) is shown (*n* = 6). Pharmacokinetics of olaparib-Ga was then analyzed by Drug Analysis System 2.0. Data are shown as the mean ± SD. (J and K) SKOV3-cis-luc xenograft tumor-bearing mice received an intravenous injection of IR780 or IR780/olaparib-Ga. The biodistribution profiles of olaparib-Ga in treated mice were examined by In Vivo Imaging System at the indicated time points postinjection (J). Tumors and main organs were obtained and examined for biodistribution quantification (K). IR780/olaparib-Ga was specifically enriched in xenograft tumors compared with IR780. Ga, gallium; FBS, fetal bovine serum; DMEM, Dulbecco’s modified Eagle’s medium; a.u., arbitrary units.

The present study explored the performance of olaparib-Ga to overcome PARPi and platinum resistance in OC cells resistant to PARPi and platinum. The olaparib-Ga nanodrug was prepared on the basis of our previous description [40]. Briefly, the bovine serum albumin (BSA) protein was used to react with Ga^3+^ to form the BSA-Ga^3+^ complex [41]. Gallic acid molecule was then added to react with BSA-Ga^3+^ complex via the formation of phenolate carboxylate group–Ga^3+^ coordination bonds to form the nanoformulation [42]. Finally, the BSA was utilized to connect olaparib via the hydrophobic effect. Meanwhile, the BSA was also served as a stabilizer to form the nanodrug. As shown in Fig. 1A, olaparib-Ga possesses uniform spherical morphology with a diameter of 6 to 8 nm.

Because of the mineralized BSA-based preparation method, the olaparib-Ga nanodrug exhibited high stability in various solutions. Dynamic light scattering results exhibited slight changes in particle size when the olaparib-Ga was dispersed in ultrapure water (water), phosphate-buffered saline (PBS), fetal bovine serum, and Dulbecco’s modified Eagle’s medium, indicating the good stability. The particle size was ~13, ~20, ~13, and ~15 nm in its hydrodynamic diameter, respectively. Meanwhile, the corresponding polydispersity index values were ~0.246, ~0.292, ~0.279, and ~0.263 (Fig. 1B). Because BSA protein was utilized as a template agent for nanostructure formation, the conformational change of BSA was an important factor [43–45]. The circular dichroism curves of BSA-Ga and olaparib-Ga were similar to that of pure BSA (Fig. S1A), demonstrating that the secondary structure of BSA was not changed during the nanodrug preparation.

The zeta potential results (Fig. 1C) showed that the olaparib-Ga nanodrug was slightly positively charged because of the loading of the negatively charged olaparib molecule. As shown in Fig. 1D, the x-ray photoelectron spectroscopy (XPS) spectra of olaparib, BSA-Ga, and olaparib-Ga nanodrug confirmed the composition of the olaparib-Ga nanodrug. The peaks containing O 1s, C 1s, and N 1s were attributed to the organic components in all 3 groups. The characteristic F 1s element attributed to the olaparib drug was observed in the olaparib-Ga group (Fig. 1E and F). Meanwhile, the Ga 3d peaks were remarkable in BSA-Ga and olaparib-Ga groups (Fig. S1B). Furthermore, the Raman spectrum was also used to investigate the olaparib combination (Fig. 1G) [46]. The clear Raman peaks around 524, 575, 649, 711, 1,025, 1,193, 1,343, and 1,445 cm^−1^ were observed in olaparib molecule, but there are almost no overlapped peaks in BSA-Ga. Meanwhile, the olaparib-Ga exhibited similar diminished characteristic peaks with the pure olaparib molecule, suggesting the successful loading of the olaparib. Therefore, the final assemblies contain all the components and confirm the correct assembly of the nanoparticle.

Subsequently, the distribution of olaparib-Ga in SKOV3-cis and A2780-cis OC cells was first confirmed by scanning electron microscopy (SEM) (Fig. 1H). Elemental mapping revealed significantly increased Ga^3+^ signals in SKOV3-cis and A2780-cis cells treated with olaparib-Ga (Fig. 1H). The cellular uptake properties of olaparib-Ga in these OC cells were monitored. After incubating IR780/olaparib-Ga with SKOV3-cis cells for 6 h, the majority of IR780/olaparib-Ga efficiently entered the cytoplasm of the cells. After 12 h of treatment, part of IR780/olaparib-Ga also entered the nuclei of the cells. Similar results were found in A2780-cis cells (Fig. S2).

Next, the present study examined the pharmacokinetics of olaparib-Ga in healthy mice. After intravenous administration of olaparib-Ga nanodrug containing 250 μg/ml of Ga^3+^, the vein blood was drawn at the indicated time points. The concentration of Ga^3+^ was then examined by inductively coupled plasma mass spectroscopy at the indicated time points (Fig. 1I). Pharmacokinetics was then evaluated using Drug Analysis System 2.0. The half-life (*t*_1/2_) of blood circulation of olaparib-Ga nanodrug was ~0.67 h (Fig. 1I). Furthermore, the biodistribution profiles of olaparib-Ga were estimated in luciferase SKOV3-cis-derived xenograft models. The SKOV3-cis-luc xenograft-bearing mice received intravenous injections of IR780 or IR780/olaparib-Ga. The treated mice were imaged by the In Vivo Imaging System at the indicated time points postinjection (Fig. 1J and Fig. S1C), and the tumors and main organs were then obtained for biodistribution quantification. The olaparib-Ga-treated group showed a higher tumor accumulation than the IR780-treated group (Fig. 1K and Fig. S1D).

### Olaparib-Ga resensitizes platinum-resistant OC cells to PARPi

**Fig. 2.**
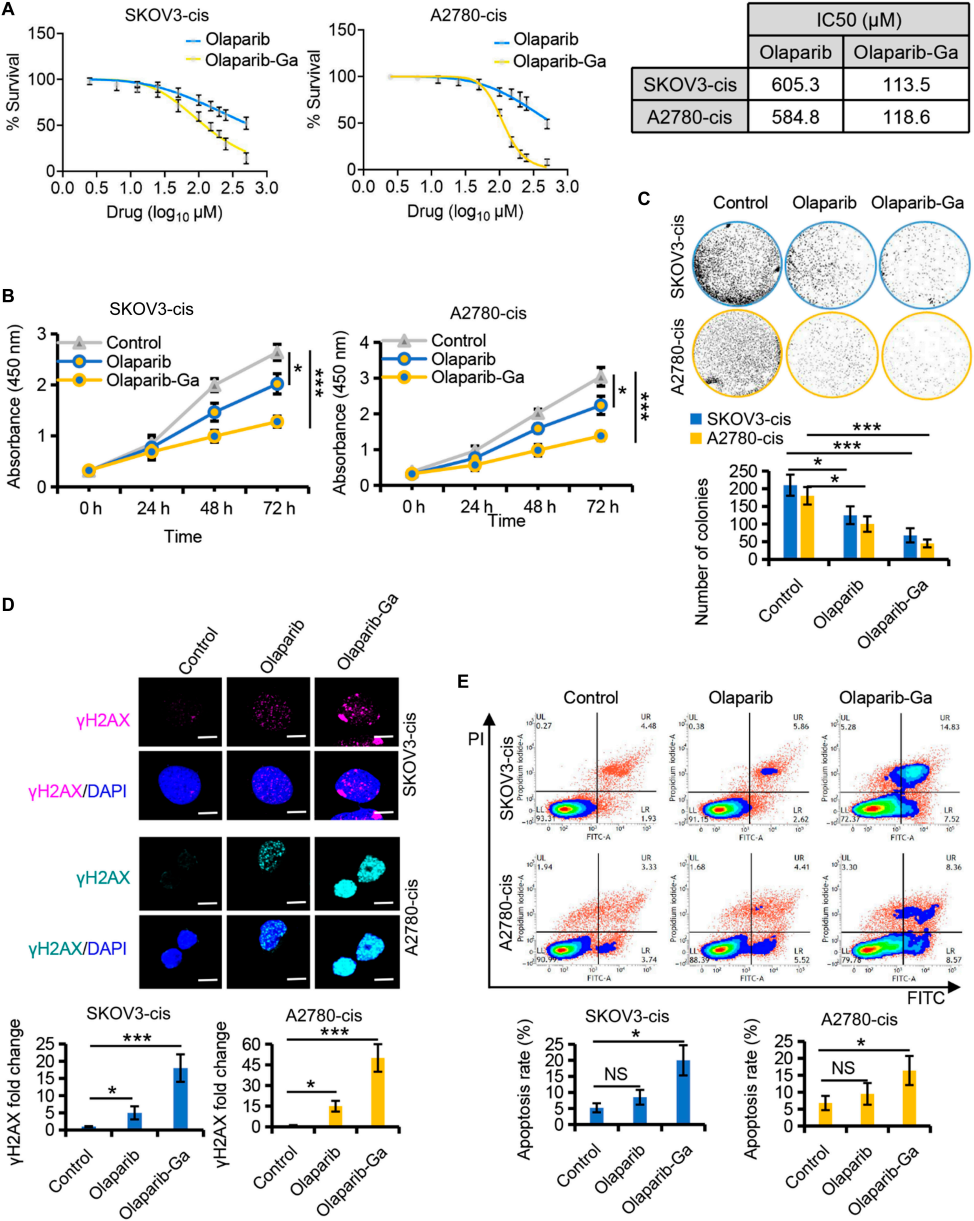
Olaparib-Ga resensitizes platinum-resistant ovarian cancer (OC) cells to olaparib. (A) Dose–response curves of olaparib [a poly (ADP-ribose) polymerase (PARP) inhibitor] and olaparib-Ga in SKOV3-cis and A2780-cis cells. The IC50 of each drug was determined. (B) Cell viabilities of platinum-resistant OC cells treated for 24, 48, and 72 h with olaparib or olaparib-Ga, as analyzed by Cell Counting Kit-8 (CCK-8) assay. (C) Colony formation assay and quantitation in the continuous presence of olaparib or olaparib-Ga in platinum-resistant OC cells. (D) Immunofluorescence images and quantitation showing γH2AX levels following different treatments. Scale bars, 10 μm. (E) Platinum-resistant OC cells treated with olaparib or olaparib-Ga were collected and stained with annexin-V-fluorescein isothiocyanate (FITC) and propidium iodide (PI). Apoptosis profiles were then determined by flow cytometry. The X-axis indicates annexin-V-FITC; the Y-axis indicates PI. *P < 0.05, and ***P < 0.001 versus the respective control. NS, not significant.

The present study evaluated the cytotoxicity of olaparib-Ga and olaparib against platinum-resistant OC cells (Fig. 2A). The half-maximum inhibitory concentration (IC50) of olaparib was ~605.3 μM in SKOV3-cis cells and ~584.8 μM in A2780-cis cells, indicating a PARPi-resistant phenotype in the 2 cell lines. The IC50 of olaparib-Ga decreased to 113.5 μM in SKOV3-cis cells and 118.6 μM in A2780-cis cells, confirming the sensitivity of these olaparib and platinum-resistant OC cells to olaparib-Ga. The cytotoxicity of olaparib-Ga was further assessed at different treatment times. Higher cell viability inhibition was observed in SKOV3-cis and A2780-cis cells treated with olaparib-Ga compared with that of cells treated with olaparib in a time-dependent manner (Fig. 2B). Olaparib-Ga also decreased the colony formation ability more than olaparib did in both resistant OC cell lines (Fig. 2C).

Because the accumulation of DNA damage is the main mechanism of olaparib-Ga, this hypothesis was investigated in resistant OC cells. The DNA double-strand break (DSB) marker γH2AX was examined. As expected, γH2AX expression significantly increased with olaparib-Ga treatment in both SKOV3-cis and A2780-cis cells, indicating the presence of DSBs (Fig. 2D). The accumulation of DNA DSBs led to significantly increased levels of apoptosis in the olaparib-Ga treatment group (Fig. 2E). Taken together, olaparib-Ga decreased the viability of PARPi/platinum-resistant OC cells via DNA damage-induced apoptosis.

### Olaparib-Ga retains SKOV3-cis tumor growth in vivo

**Fig. 3.**
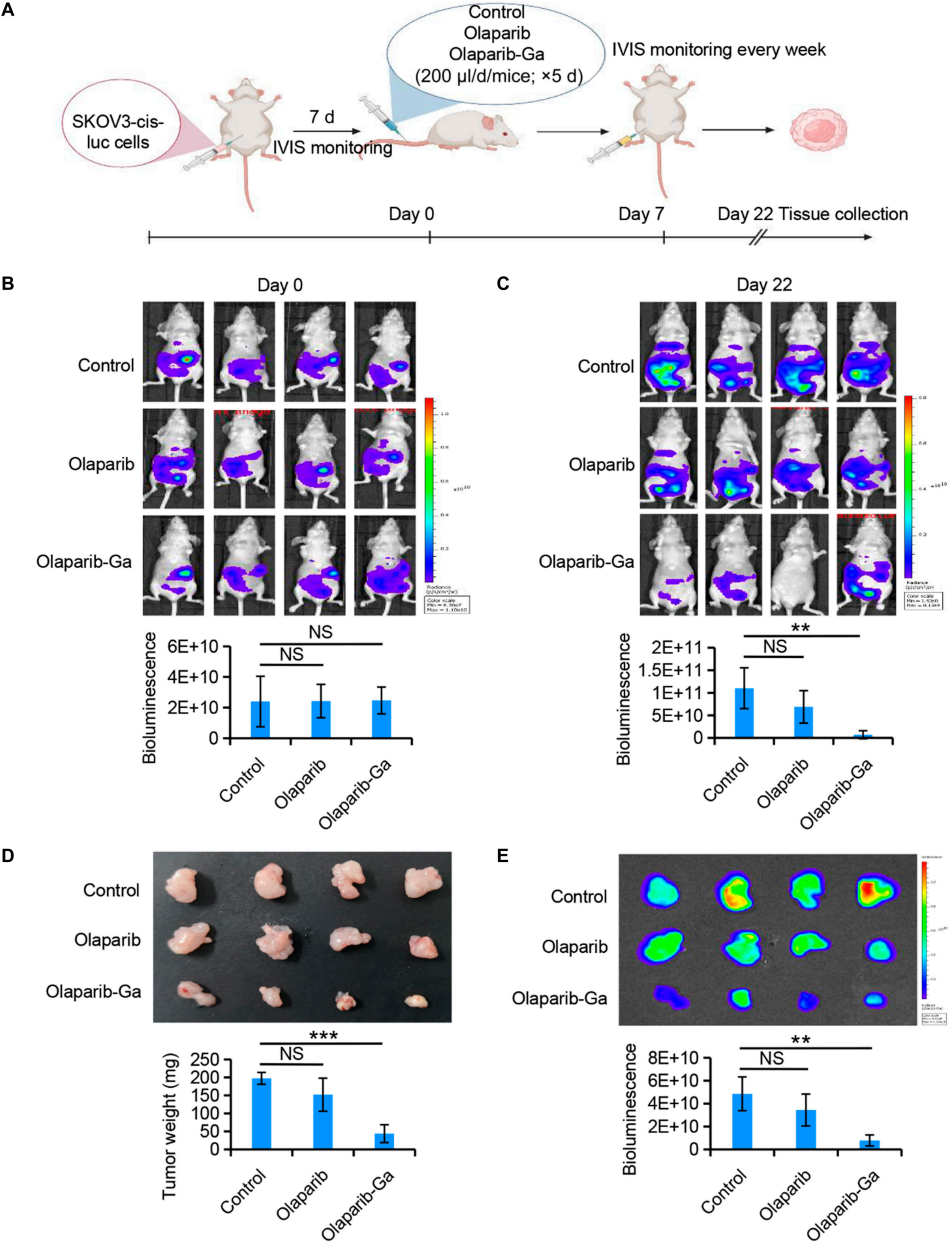
Olaparib-Ga inhibits tumor growth in vivo. (A) Workflow showing the experimental process of olaparib-Ga-mediated tumor growth inhibition in SKOV3-cis-luc-derived xenograft mice. (B) Fluorescent images of luciferase SKOV3-cis xenograft-bearing mice subjected to the indicated treatments (*n* = 4 per grou; 3 groups). Treatment groups: (a) control (PBS): 200 μl/mouse/d intravenously (IV) for 5 d; (b) olaparib 0.5 mM, 200 μl/mouse/d IV for 5 d; and (c) olaparib-Ga 0.5 mM, 200 μl/mouse/d IV for 5 d. Tumor growth was monitored weekly via In Vivo Imaging System (IVIS). Representative bioluminescence images of SKOV3-cis-luc xenograft-bearing mice at days 0 and 22. (D and E) Tumor photographs (D), tumor weight (D), and bioluminescence images (E) of luciferase SKOV3-cis-derived xenograft tumors. **P < 0.01 and ***P < 0.001 versus the respective control.

The current study next determined whether olaparib-Ga could suppress PARPi/platinum-resistant tumor growth in vivo (Fig. 3A). SKOV3-cis tumors treated with olaparib-Ga were significantly inhibited compared with those treated with control and olaparib in mouse xenograft models (Fig. 3B to E). The results of immunohistochemical (IHC) staining also confirmed the decreased level of Ki-67 in tumors subject to olaparib-Ga treatment (Fig. S3).

To evaluate the side effects of olaparib-Ga, blood routine and biochemistry analyses were performed (Fig. S4A). The hematological parameters were all within the normal range on day 22 after the first administration of olaparib-Ga. No obvious histological toxicity was observed in the main organs (Fig. S4B). In addition, the noncancerous ovarian cell line IOSE-80 was used to confirm the side effects of olaparib-Ga. The cell viability curves showed that the IC50 of olaparib-Ga was not significantly decreased compared with that of olaparib in this cell line, indicating that olaparib-Ga did not increase the effect of olaparib on normal ovarian cell viability (Fig. S5). These data indicated effective antitumor performance and good biocompatibility of olaparib-Ga in the treatment of resistant OC tumors.

### Olaparib-Ga increases the sensitivity of platinum-resistant OC cells to cisplatin and carboplatin

**Fig. 4.**
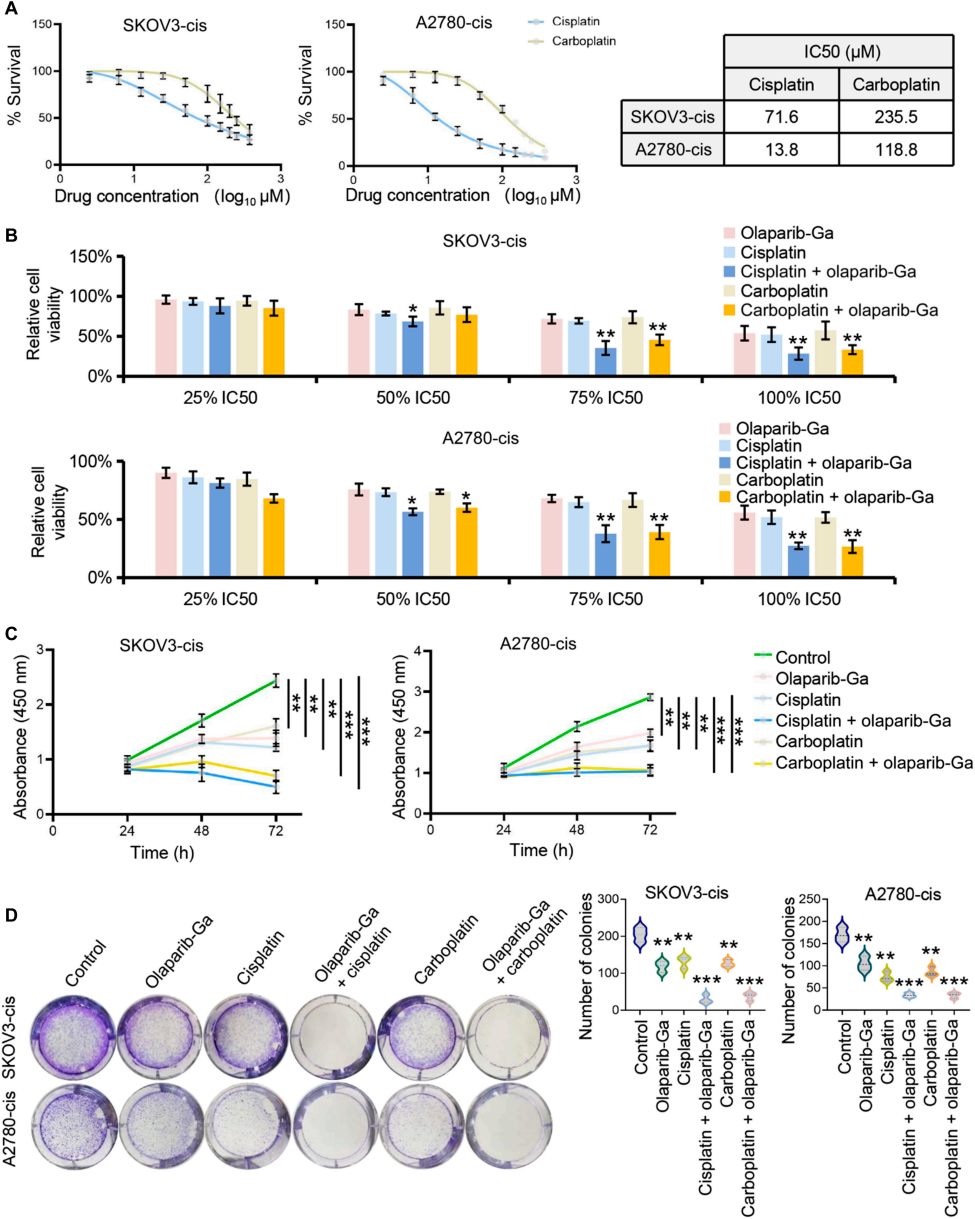
Olaparib-Ga increases the sensitivity of platinum-resistant OC cells to cisplatin and carboplatin. (A) IC50 of cisplatin and carboplatin in SKOV3-cis and A2780-cis cells. The IC50 values were determined according to dose–response curves using GraphPad Prism 9.2 software. Data are shown as the mean ± SD. (B) Cell viability analysis for the combination of olaparib-Ga and cisplatin or carboplatin. Cells were treated with the indicated concentrations of olaparib-Ga and cisplatin or carboplatin for 48 h. Cell viability was detected using a CCK-8 kit. (C) Cells were treated with olaparib-Ga or cisplatin, a combination of olaparib-Ga and cisplatin, carboplatin, or a combination of olaparib-Ga and carboplatin using 75% of the IC50 value of each drug. Cell viability was determined by time-lapse imaging for 72 h in the continuous presence of the indicated treatments using a CCK-8 kit. (D) Colony formation assay and quantitation in the presence of the indicated drugs at 7.5% of IC50 doses in SKOV3-cis and A2780-cis cells. *P < 0.05, **P < 0.01, and ***P < 0.001.

Considering the cytotoxicity of olaparib-Ga in platinum-resistant OC cells, the current study examined whether combining olaparib-Ga with cisplatin or carboplatin would exacerbate the cytotoxicity more than that of olaparib-Ga alone. The IC50 values of cisplatin and carboplatin in SKOV3-cis and A2780-cis cells were as follows: SKOV3-cis: 71.6 μM cisplatin and 235.5 μM carboplatin; and A2780-cis: 13.8 μM cisplatin and 118.8 μM carboplatin (Fig. 4A). These cells were treated with 4 different doses of olaparib-Ga, cisplatin, or carboplatin at 25%, 50%, 75%, or 100% of IC50 or with a combination of olaparib-Ga and cisplatin or carboplatin. The co-treatment of SKOV3-cis cells with olaparib-Ga and cisplatin or carboplatin at 75% and 100% IC50 significantly suppressed cell viability compared with the effects of each individual treatment (Fig. 4B). Similar results were found in A2780-cis cells (Fig. 4B). To minimize drug toxicity, ~75% of the IC50 value of each drug (namely, SKOV3-cis: 85 μM olaparib-Ga, 55 μM cisplatin, and 175 μM carboplatin; and A2780-cis: 90 μM olaparib-Ga, 10 μM cisplatin, and 90 μM carboplatin) was next selected for combination study. It was determined that both SKOV3-cis and A2780-cis cells showed increased sensitivities to the combination of olaparib-Ga and cisplatin or carboplatin compared with that of each treatment alone, as shown by the suppressed cell viability (Fig. 4C) and colony formation ability (Fig. 4D).

### Combination of olaparib-Ga with cisplatin or carboplatin induces DNA damage and activates the ATM/ATR–Chk1/Chk2 signaling pathway

**Fig. 5.**
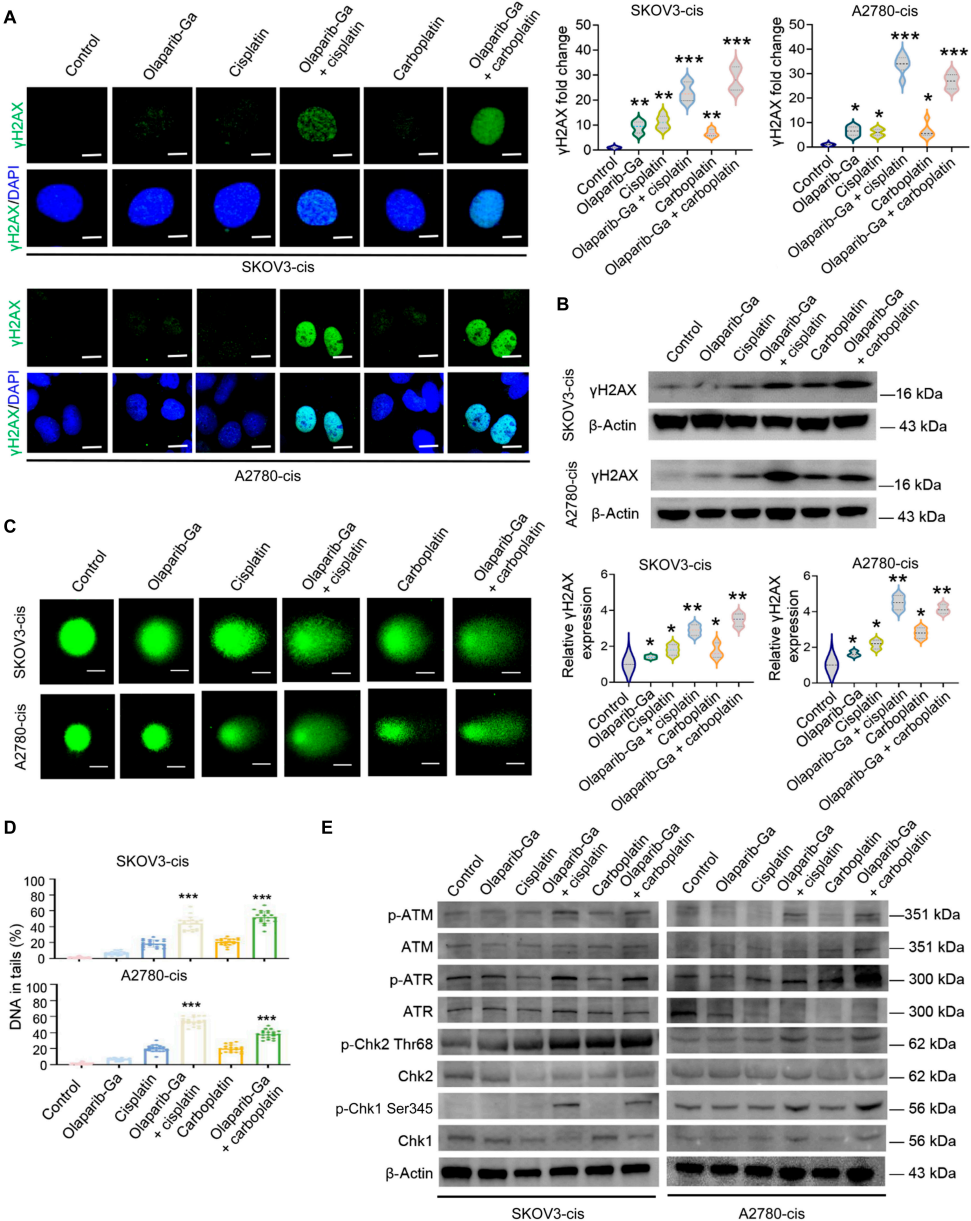
Combination of olaparib-Ga with cisplatin or carboplatin induces DNA damage and activates the ATM/ATR–Chk1/Chk2 signaling pathways in SKOV3-cis and A2780-cis cells. (A and B) Immunofluorescence images (A) and Eestern blotting analysis (B) of cells treated with olaparib-Ga or cisplatin, a combination of olaparib-Ga and cisplatin, carboplatin, or a combination of olaparib-Ga and carboplatin using 75% of the IC50 of each drug, showing γH2AX expression after 48 h of exposure. Scale bars, 10 μm. (C and D) Comet assay of cells exposed to the indicated treatments in (A) and (B) for 48 h. Scale bars, 20 μm. The average percentage of DNA in tails was calculated from ≥20 cells in each group. (E) Western blotting of ATM, ATR, Chk1, Chk2, p-ATM, p-ATR, p-Chk1, and p-Chk2 in SKOV3-cis and A2780-cis cells following 48 h of exposure to olaparib-Ga or cisplatin, a combination of olaparib-Ga and cisplatin, carboplatin, or a combination of olaparib-Ga and carboplatin using 75% of the IC50 of each drug. *P < 0.05, **P < 0.01, and ***P < 0.001. ATM, ataxia telangiectasia mutated; ATR, AMT- and Rad3-related; Chk, checkpoint kinase; p-, phosphorylated.

Because DNA damage is involved in the mechanism of action of chemotherapeutic agents such as cisplatin and carboplatin [47,48], the present study next evaluated whether olaparib-Ga and cisplatin or carboplatin could increase the effects of DNA damage. As expected, it was found that γH2AX expression levels were slightly upregulated in single drug-treated SKOV3-cis and A2780-cis cells and further significantly increased in the co-treated cells, according to the results of immunofluorescence and Western blotting (Fig. 5A and B and Fig. S6). Moreover, following the co-treatment of olaparib-Ga and cisplatin or carboplatin, comet assay showed elevated DNA damage (Fig. 5C and D).

ATM and ATR are the first steps in the response to DNA damage [49]. ATM and ATR activate the phosphorylation of the cell cycle checkpoint genes Chk1 and Chk2 to block cell cycle progression [50]. The present study evaluated whether the combination of olaparib-Ga and cisplatin or carboplatin could activate the ATM/ATR–Chk1/Chk2 signaling pathways more than the drug(s) alone could do. Olaparib-Ga, cisplatin, or carboplatin alone did not significantly increase the expression levels of ATM, ATR, Chk1, Chk2, phosphorylated (p)-ATM, p-ATR, p-Chk1, or p-Chk2 in SKOV3-cis or A2780-cis cells (Fig. 5E and Fig. S7). Although the total protein expression were not significantly changed, co-treatment with olaparib-Ga and cisplatin or carboplatin significantly increased the phosphorylated levels of ATM, ATR, Chk1, and Chk2 expression in both resistant cell lines (Fig. 5E and Fig. S8). These results suggested that co-treatment with olaparib-Ga and cisplatin or carboplatin could cause DNA damage response through activation of the ATM/ATR–Chk1/Chk2 signaling pathways.

### Olaparib-Ga in combination with cisplatin or carboplatin arrests cell cycle at S and G_2_/M phases and increases apoptosis

**Fig. 6.**
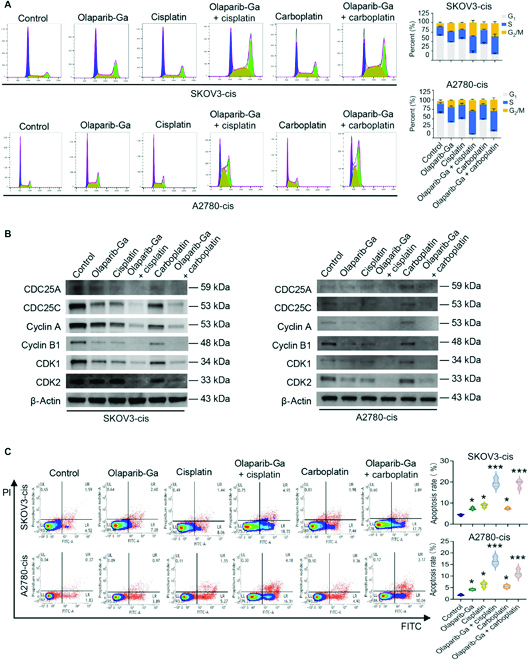
Olaparib-Ga in combination with cisplatin or carboplatin arrests the cell cycle at the S and G_2_/M phases and increases apoptosis in SKOV3-cis and A2780-cis cells. (A) Cells were treated with olaparib-Ga or cisplatin, a combination of olaparib-Ga and cisplatin, carboplatin, or a combination of olaparib-Ga and carboplatin using 75% of the IC50 of each drug, and their cell cycle distribution was evaluated. (B) Combination of olaparib-Ga and cisplatin or carboplatin reduced the protein levels of CDC25A, CDC25C, cyclin A, cyclin B1, CDK1, and CDK2 in SKOV3-cis and A2780-cis cells. (C) The apoptotic rate of SKOV3-cis and A2780-cis cells subjected to the indicated treatments is shown. *P < 0.05, and ***P < 0.001.

The activation of the cell cycle checkpoint transducer kinases Chk1 and Chk2 can block cell cycle progression at the S or G_2_/M phases through inducing the proteosomal degradation of the CDC25 family and subsequently inhibiting the activity of CDKs/cyclins [49,50]. The current study next examined cell cycle progression in response to co-treatment with olaparib-Ga and cisplatin or carboplatin. Of note, drug co-treatment could significantly arrest the cell cycle at the S and G_2_/M phases compared with the effect of olaparib-Ga, cisplatin, or carboplatin alone or control in both SKOV3-cis and A2780-cis cells (Fig. 6A). The critical target of the S checkpoint is cyclin A/CDK kinase, whose activation is inhibited by CDC25A [51]. The G_2_/M checkpoint suppresses the activity of CDC25C/cyclin B/CDK1 [50]. The present study verified the expression levels of CDC25A, cyclin A, CDK2, CDC25C, cyclin B, and CDK1 to further understand the mechanism of cell cycle arrest for co-treatments. As expected, we confirmed that the protein expression levels of CDC25A, cyclin A, CDK2, CDC25C, cyclin B, and CDK1 were all significantly inhibited in response to combination of olaparib-Ga and cisplatin or carboplatin in both cell lines (Fig. 6B and Fig. S9). Next, the current study examined whether the accumulation of DNA damage and arrested cell cycle progression observed with olaparib-Ga and cisplatin or carboplatin co-treatment would precede increased levels of apoptosis. Single-drug treatment caused a slight increase in apoptosis compared with that of the control group. Co-treatment with olaparib-Ga and cisplatin or carboplatin caused a significantly higher increase in apoptosis (Fig. 6C). Altogether, these results indicated that co-treatments could cause severe DNA damage, arrest cell cycle, and ultimately lead to apoptosis, thus inducing cell death.

### Olaparib-Ga recovers chemosensitivity of platinum-resistant OC tumors to cisplatin and carboplatin in mice xenograft models

**Fig. 7.**
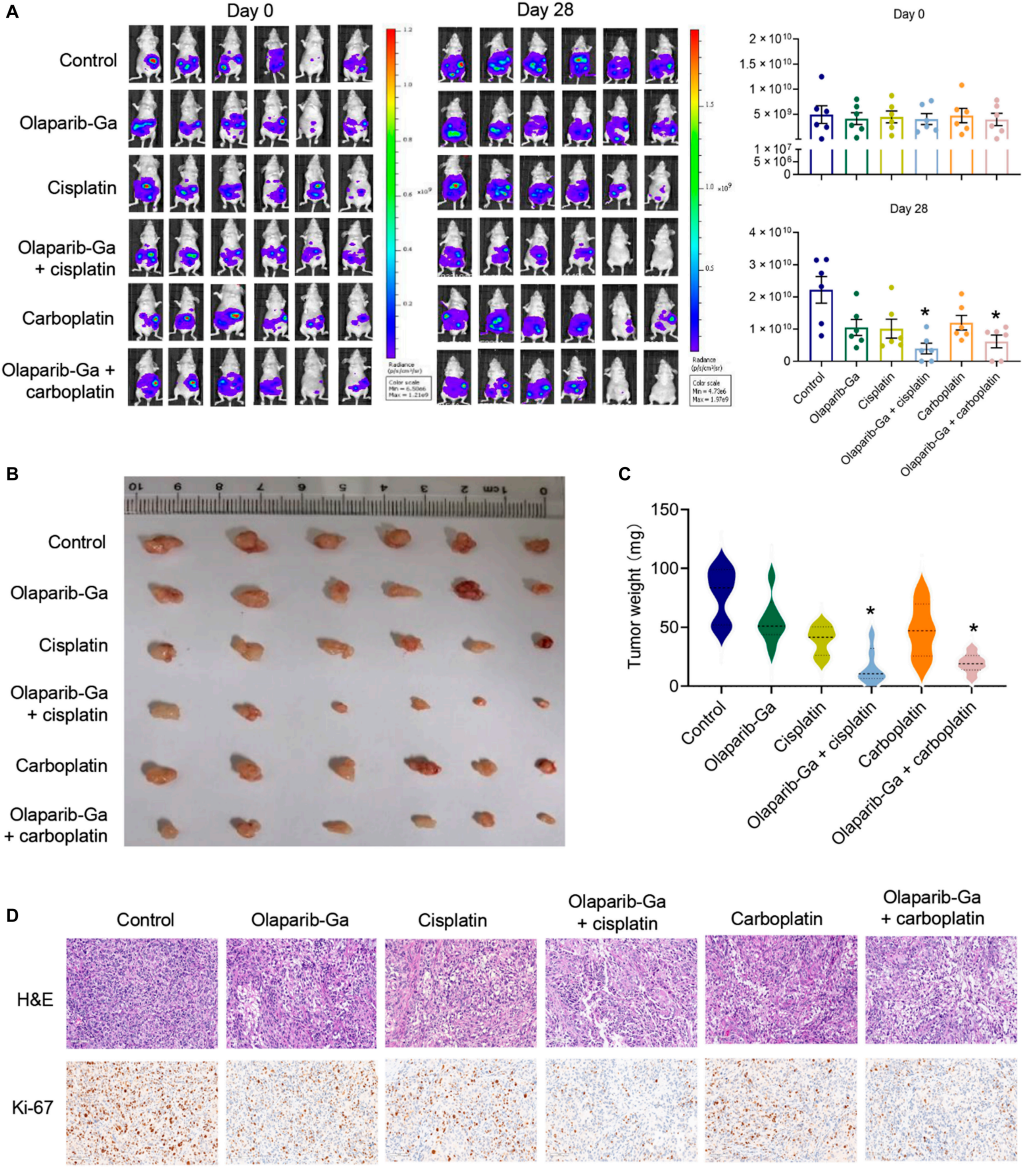
Olaparib-Ga recovers the chemosensitivity of platinum-resistant OC tumors to cisplatin and carboplatin in mouse xenograft models. (A) Fluorescent images of luciferase SKOV3-cis xenograft-bearing mice subjected to the indicated treatments (*n* = 6 per group; 3 groups). Treatment groups: (a) control (PBS; 200 μl/mouse/d for 3 d), (b) olaparib-Ga (0.5 mM; 200 μl/mouse/d for 3 d), (c) cisplatin (4 mg/kg per mouse for 1 d), (d) co-treatment of olaparib-Ga (0.5 mM; 200 μl/mouse/d for 3 d) and cisplatin (4 mg/kg per mouse for 1 d), (e) carboplatin (30 mg/kg per mouse for 1 d), and (f) co-treatment of olaparib-Ga (0.5 mM; 200 μl/mouse/d for 3 d) and carboplatin (30 mg/kg per mouse for 1 d). Tumor growth was determined weekly using In Vivo Imaging System. Representative bioluminescence images of SKOV3-cis-luc xenograft-bearing mice at days 0 and 28. (B and C) Tumor image (B) and weight (C) of luciferase SKOV3-cis-derived xenograft tumors subjected to the indicated treatments. (D) Hematoxylin and eosin (H&E) staining and Ki-67 immunohistochemical analysis of xenograft tumors after systemic treatment in each group. *P < 0.05.

Finally, the present study investigated whether the olaparib-Ga nanodrug could recover the chemosensitivity of SKOV3-cis tumors in a mouse xenograft model. Six different treatments, including PBS (control), olaparib-Ga, cisplatin, co-treatment of olaparib-Ga and cisplatin, carboplatin, and co-treatment of olaparib-Ga and carboplatin, were administered by intravenous injection to SKOV3-cis-bearing mice (*n* = 6 in each group). Olaparib-Ga was first administered once every day at a dose of 200 μl per mouse for 3 d. Cisplatin and carboplatin were administered once to each mouse at a dose of 4 and 30 mg/kg, respectively. Four weeks after the treatment, the groups with single olaparib-Ga, cisplatin, or carboplatin treatment showed limited efficacy in inhibiting tumor growth (Fig. 7A). Notably, tumor growth in both co-treatment groups was significantly suppressed compared with that of the mice treated with control, olaparib-Ga, cisplatin, or carboplatin alone (Fig. 7A). The tumor size and weight were much smaller in the 2 co-treatment groups than in the control group (Fig. 7B and C). The results of IHC staining further confirmed that co-treatments with olaparib-Ga and cisplatin or carboplatin were the most effective in suppressing tumor growth, as determined by Ki-67 staining (Fig. 7D).

**Fig. 8.**
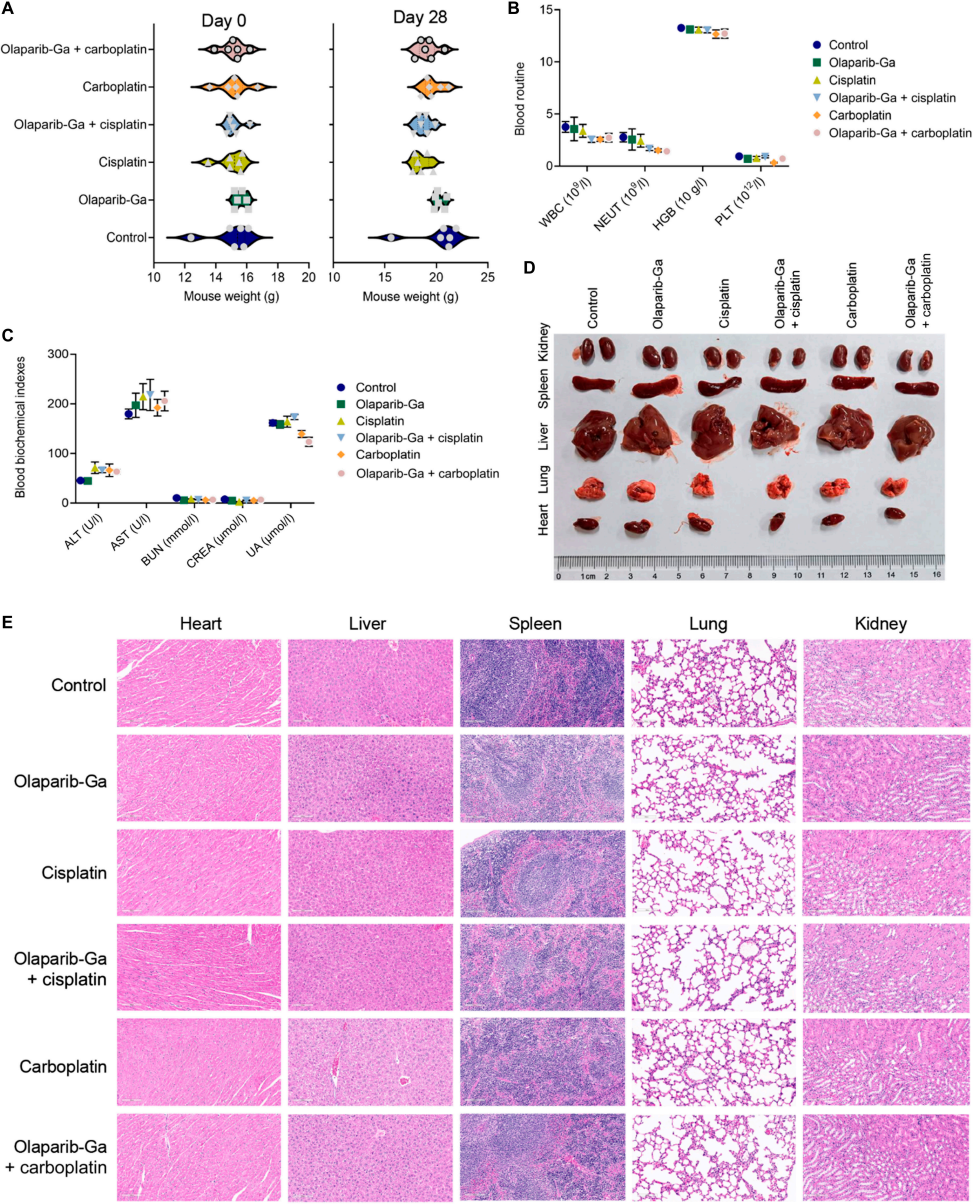
Toxicity analysis of olaparib-Ga and cisplatin or carboplatin co-treatment in mouse xenograft models. (A) The body weight at days 0 and 28 of SKOV3-cis-luc xenograft-bearing mice subjected to the indicated treatments is shown. (B and C) The indicated hematological parameters, including blood routine (B) and biochemistry (C), were examined in different treatment groups of mice. (D) Representative photographs of main organs in different treatment groups are shown. (E) Representative images of H&E staining of the aforementioned main organs in the different treatment groups are shown. WBC, white blood cell count; NEUT, neutrophil; HGB, hemoglobin; PLT, platelet count; ALT, alanine aminotransferase; AST, aspartate aminotransferase; BUN, blood urea nitrogen; CREA, creatinine; UA, uric acid.

The current study further examined whether co-treatments of olaparib-Ga and cisplatin or carboplatin caused severe systematic side effects. In the SKOV3-cis-bearing mice, the 2 co-treatment groups showed no obvious mouse body weight loss on day 28 after the first injection (Fig. 8A). Multiple hematological parameters were determined (Fig. 8B and C), and no significant difference was observed in the co-treatment groups. Moreover, hematoxylin and eosin (H&E) staining of main organ tissues, including heart, liver, spleen, lung, and kidney, showed no noticeable histological toxicity (Fig. 8D and E). In addition, healthy mice also received co-treatment with olaparib-Ga and cisplatin and co-treatment with olaparib-Ga and carboplatin (*n* = 3 in each group) (Fig. S10A). Upon treatment, mouse weight, hematological parameters, and major organs were evaluated (Fig. S10B to E). No obvious drug toxicity was observed. These results suggested the biocompatibility of the co-treatments of olaparib-Ga and cisplatin or carboplatin. Altogether, the present findings indicate that applying olaparib-Ga is a promising strategy to reverse the chemoresistance of OC.

## Discussion

Platinum, particularly cisplatin and carboplatin, represents a prominent therapeutic option in the treatment of OC. However, cancer cells develop resistance to platinum, resulting in therapeutic failure. Furthermore, platinum-resistant tumors also fail to respond to target therapy PARPi [11]. Considerable studies have been performed to restore platinum sensitivity. However, platinum resistance remains a critical goal for anticancer therapy in the clinical setting. The present study developed olaparib-Ga nanoparticles to confer PARPi resistance in platinum-resistant OC cells. Co-treatment of olaparib-Ga nanodrug and cisplatin or carboplatin could induce DNA damage, followed by the activation of the ATM/ATR–Chk1/Chk2 signaling pathway. Response to DNA damage caused cell cycle progression blockage at S and G_2_/M phases. Nonrepairable DNA damage finally elicited proapoptotic outcomes.

Our group previously developed a self-assembly olaparib-Ga nanodrug to sensitize HR-proficient OC cells to PARPi [40]. Olaparib-Ga could inhibit the proliferation of HR-proficient OC cells in an apoptosis-dependent manner via activation of the Fe^2+^/ROS/MAPK/HMOX1 and inhibition of PI3K/AKT signaling pathways. The present study investigated whether this olaparib-Ga nanodrug could also exert antitumor effects in platinum-resistant OC cells. As expected, olaparib-Ga treatment resensitized platinum-resistant SKOV3-cis and A2780-cis OC cells to olaparib. Olaparib showed limited efficiency in the treatment of platinum-resistant OC cells, while the cytotoxicity of olaparib-Ga was much higher than that olaparib alone. The olaparib-Ga nanodrug could effectively increase DNA DSBs, promote apoptosis and inhibit cell viability in platinum-resistant OC cells. Moreover, olaparib-Ga exerted efficient suppression of tumor growth in platinum-resistant OC cell-bearing mice without increased drug toxicity. These results suggested that Ga added to olaparib could restore the sensitivity to PARPi of platinum-resistant OC cells.

Platinum is mainly involved in DNA damage response and apoptosis [52,53]. The signaling transduction pathways that link platinum-induced DNA damage with apoptosis are characterized by the activation of the AMT and ATR kinases. ATM and ATR are coordinated in numerous cellular stress responses and permit a rapid and sensitive activation of cell cycle checkpoints [49]. ATM and ATR preferentially mediate the phosphorylation of Chk2 and Chk1, respectively [54]. Activated cell cycle checkpoints stop cell cycle progression and lead to apoptosis followed by cell death [55,56]. Alterations in any of these signaling pathways are often responsible for the platinum-resistant phenotype of OC cells [5]. The present findings revealed that the major signaling pathways were reactivated, and cisplatin and carboplatin sensitivity were restored in platinum-resistant OC cell lines. It was showed that combined olaparib-Ga with cisplatin or carboplatin treatment caused accumulation of DNA damage, which was accompanied by increased DNA damage response and activation of the ATM/ATR–Chk1/Chk2 signaling pathways, leading to blockage of the S and G_2_/M phases, as determined by reduced CDC25A/cyclin A/CDK2 and CDC25C/cyclin B/CDK1 expression. In contrast to maintaining cell survival and resistant genotype, the DNA damage induced by the combination of olaparib-Ga and cisplatin or carboplatin in the present study was beyond repair, and cells underwent apoptotic cell death.

The present animal experiments demonstrated that combination of olaparib-Ga with cisplatin or carboplatin was well tolerated for the durations of the co-treatment. The results showed that the above co-treatment led to a significant tumor growth suppression compared with that caused by single-drug or control treatment in platinum-resistant OC mouse models. Taken together, these results suggested a development of a chemosensitization strategy with important clinical implications.

In summary, the present study demonstrated that olaparib-Ga induced DNA damage and suppressed tumor growth in PARPi- and platinum-resistant OC cells. Combined treatment of olaparib-Ga with cisplatin or carboplatin led to increased DNA damage-induced apoptosis and cell death. Applying olaparib-Ga may provide a promising strategy to resensitize OC to platinum-based chemotherapies, which may be of significant benefit to the survival of patients with resistant OC.

## Materials and Methods

### Drugs

Olaparib (catalog no. HY-10162), cisplatin (catalog no. HY-17394), and carboplatin (catalog no. HY-17393) were purchased from MedChemExpress. Ga (III) (catalog no. G828121), gallic acid (catalog no. G823163), and BSA (catalog no. A801320) were purchased from Shanghai Macklin Biochemical Co. Ltd.

### Cell cultures

SKOV3-cis and A2780-cis platinum-resistant OC cells and IOSE-80 ovarian surface epithelial cells were purchased from American Type Culture Collection and cultured according to standard protocols.

### Preparation of olaparib-Ga

The olaparib-Ga nanodrug was synthesized according to our previous report [40], using BSA, Ga, gallic acid, and olaparib.

### Characterization of olaparib-Ga nanodrug

Composition and elemental analyses were performed by x-ray photoelectron spectroscopy (K-Alpha; Thermo Fisher Scientific Inc.). The protein structure was investigated by circular dichroism (JASCO J-1500; JASCO Corporation).

### Scanning electron microscopy (SEM)

Cells were treated with olaparib-Ga for 48 h, and then fixed, washed, and dehydrated with a graded series of ethanol and finally tert-butanol. After drying, cells were coated with platinum and assessed by SEM (FEI Nova NanoSEM 450; Thermo Fisher Scientific Inc.). The Ga^3+^ elements of the samples were examined using energy-dispersive x-ray spectroscopy (Octane EDS-70; EDAX; Ametek Inc.).

### Evaluation of cellular uptake of olaparib-Ga

Cells were incubated with IR780-labeled olaparib-Ga nanodrug for 1, 3, 6, or 12 h and washed with PBS. The nuclei were stained with 4′,6-diamidino-2-phenylindole (DAPI), and the treated cells were imaged under a confocal laser scanning microscope (FLUOVIEW FV1200; Olympus Corporation). Red fluorescence indicated IR780-labeled olaparib-Ga and blue fluorescence indicated DAPI-labeled nuclei.

### Pharmacokinetics study

Healthy female BALB/c nude mice were intravenously injected with olaparib-Ga (0.5 mM, 200 μl dose per mouse; *n* = 6). At predetermined time intervals, 20-μl orbital vein blood was obtained. The concentrations of Ga^3+^ were [44] evaluated by inductively coupled plasma mass spectroscopy. Pharmacokinetics was determined using Drug Analysis System 2.0 (BioGuider).

### Biodistribution

Tumor-bearing female BALB/c nude mice were randomly divided into 2 groups (*n* = 3 each group) and intravenously injected with either naked IR780 or IR780/olaparib-Ga nanodrug. The mice were imaged using the In Vivo Imaging System (PerkinElmer Inc.) at the indicated time points. Tumors and major organs were then harvested and imaged.

### Cell viability assay

Cells (5 × 10^3^ cells per well) were plated in 96-well plates. At the indicated time point, cells were cultured with 10% Cell Counting Kit-8 (CCK-8) solution (Dojindo Laboratories Inc.). The absorbance in each well was measured at 495 nm using a spectrophotometer (Thermo Fisher Scientific Inc.).

### Colony formation assay

In total, 1 × 10^3^ cells were seeded in a 6-well plate and cultured as indicated for 10 d. The colonies were fixed with 4% formaldehyde, stained with 2% crystal violet for 5 min, and visualized using a camera (Canon Inc.).

### Immunofluorescence

Treated cells treated as indicated were fixed with 4% formaldehyde, permeabilized with 0.3% Triton X-100, blocked with 10% fetal bovine serum, and incubated with a primary anti-γH2AX antibody (Abcam) overnight at 4 °C. Cells were washed in PBS 3 times, incubated with Alexa Fluor Plus 488-labeled secondary antibody (Invitrogen; Thermo Fisher Scientific Inc.), stained with DAPI (Abcam), and examined with a confocal laser scanning microscope (FLUOVIEW FV1200; Olympus Corporation).

### Flow cytometry

Cell cycle and apoptosis were analyzed by flow cytometry. For apoptosis, cells treated as indicated were obtained and incubated with annexin IV and propidium iodide staining solution (Multi Sciences) for 30 min and analyzed using a flow cytometer (FACSVerse; BD Biosciences). For cell cycle analysis, cells were collected, washed twice with PBS, stained with propidium iodide (Multi Sciences) for 1 h, and analyzed using the aforementioned flow cytometer. Data were processed with FlowJo v10 (FlowJo LLC).

### Immunoblotting

Protein lysates were resolved by 4 to 20% sodium dodecyl sulfate-polyacrylamide gel electrophoresis (GenScript) and then electroblotted onto polyvinylidene difluoride membranes (Bio-Rad Laboratories Inc.). After blocking, membranes were incubated with primary antibodies against γH2AX (Abcam, 1:5,000), ATM (Huabio, 1:1,000), p-ATM (Huabio, 1:1,000), ATR (Huabio, 1:1,000), p-ATR (Proteintech, 1:1,000), Chk1 (Proteintech, 1:1,000), p-Chk1 (Proteintech, 1:1,000), p-Chk2 (Proteintech, 1:1,000), CDC25A (Huabio, 1:1,000), cyclin A (Huabio, 1:1,000), CDK2 (Huabio, 1:1,000), CDC25C (Proteintech, 1:1,000), cyclin B1 (Proteintech, 1:1,000), CDK1 (Proteintech, 1:1,000), and β-actin (Proteintech, 1:3,000). Immunoblotting was determined following standard procedures. The data were representative 3 independent experiments.

### Comet assay

Comet assay was conducted using a Comet Assay Kit (Abcam, catalog no. ab238544). Briefly, cells treated as indicated were harvested and resuspended at a density of 1 × 10^5^ cells/ml in PBS. Under low lighting, samples were combined with comet agarose and spread onto a precoated base layer. Slides were immersed into lysis buffer in the dark at 4 °C for 1 h and transferred to an electrophoresis chamber for 30 min. The slides were then immersed in distilled H_2_O, followed by 70% ethanol. Upon air drying, Vista Green DNA Dye was added for 15 min. Images were acquired by epifluorescence microscopy (CTR6500; Leica Microsystems Inc.), and DNA damage was analyzed by CometScore 2.1 (TriTek Corp).

### Animal experiments

Female BALB/c nude mice (5 to 6 weeks old) were purchased from Shanghai LAC Laboratory Animal Co. Ltd. The animal study was approved by the Institutional Animal Care and Use Committee of Zhejiang Chinese Medical University (approval no. IACUC-20210913-04). Mice were bred in specific pathogen-free facility with suitable temperature and humidity. Each mouse was administered an intraperitoneal injection of 5 × 10^6^ SKOV3-cis-luc cells. At 1 week postinjection, SKOV3-cis-luc tumor-bearing mice were randomly divided into 2 groups. In the first group, mice were intravenously injected with (a) 200 μl of PBS, (b) 200 μl of olaparib (0.5 mM), or (c) 200 μl of olaparib-Ga (0.5 mM with respect to olaparib) daily for 5 d. In the second group, mice were intravenously injected with (a) PBS (200 μl/d for 3 d), (b) 0.5 mM olaparib-Ga (200 μl/d for 3 d), (c) 4 mg/kg of cisplatin (once), (d) co-treatment of 0.5 mM olaparib-Ga (200 μl/d for 3 d) and 4 mg/kg of cisplatin (once), (e) 30 mg/kg of carboplatin (once), or (f) co-treatment of 0.5 mM olaparib-Ga (200 μl/d for 3 d) and 30 mg/kg of carboplatin (once). The animals were euthanized after 4 weeks of their first drug injection. Hematology tests were conducted. The tumors and main organs were harvested and subjected to H&E and IHC staining.

Healthy female BALB/c mice of 5 to 6 weeks of age were randomly divided into 3 groups (*n* = 3) and administered intravenous injections of either PBS, olaparib-Ga (daily for 3 d) and cisplatin (once), or olaparib-Ga (daily for 3 d) and carboplatin (once) at the indicated dose. Mouse weight was determined postinjections, and blood samples were collected for hematological examination. Organs were obtained 24 h after the final injection and subjected to H&E staining.

### Statistical analysis

Statistical analyses were performed using GraphPad Prism 9.2 software (GraphPad Software; Dotmatics). Student’s *t* test and one-way analysis of variance were used to test the differences between treatments. The data are shown as the mean ± SD of 3 independent experiments. *P* < 0.05 was considered to indicate a statistically significant difference.

## Data Availability

All study data are included in the article and supporting information.

## Supplementary Materials

Supplementary Materials Fig. S1. Features of olapairb-Ga, relative to Fig. 1. Fig. S2. Cellular uptake performance of olaparib-Ga in SKOV3-cis and A2780-cis cells, relative to Fig. 1. Fig. S3. Olaparib-Ga inhibits Ki-67 expression of SKOV3-cis-derived xenograft tumors, related to Fig. 3. Fig. S4. Olaparib-Ga does not induce severe toxicity of SKOV3-cis-derived xenograft mice, related to Fig. 3. Fig. S5. Olaparib-Ga does not induce severe toxicity in human normal ovarian cell lines, related to Fig. 3. Fig. S6. The original gel images of Fig. 5B. Fig. S7. The original gel images of Fig. 5E. Fig. S8. Combination of olaparib-Ga with cisplatin or carboplatin activates ATM/ATR-Chk1/Chk2 pathways in SKOV3-cis and A2780-cis cells, related to Fig. 5. Fig. S9. Olaparib-Ga in combination with cisplatin or carboplatin blocks cell cycle progression at S and G_2_/M phases in SKOV3-cis and A2780-cis cells, related to Fig. 6. Fig. S10. Co-treatment of olaparib-Ga and cisplatin or carboplatin does not cause noticeable hematologic and histological toxicity in healthy mice, related to Fig. 8.
