# Supplementary material for: Nanoengineered Gallium Ion Incorporated Formulation for Safe and Efficient Reversal of PARP Inhibition and Platinum Resistance in Ovarian Cancer
Source: Research (Wash D C). 2023 Mar 9;6:0070. doi: 10.34133/research.0070 (PMC10013963; doi:10.34133/research.0070)
Supplement: Supplementary Materials — Fig. S1. Features of olapairb-Ga, relative to Fig. 1. Fig. S2. Cellular uptake performance of olaparib-Ga in SKOV3-cis and A2780-cis cells, relative to Fig. 1. Fig. S3. Olaparib-Ga inhibits Ki-67 expression of SKOV3-cis-derived xenograft tumors, related to Fig. 3. Fig. S4. Olaparib-Ga does not induce severe toxicity of SKOV3-cis-derived xenograft mice, related to Fig. 3. Fig. S5. Olaparib-Ga does not induce severe toxicity in human normal ovarian cell lines, related to Fig. 3. Fig. S6. The original gel images of Fig. 5B. Fig. S7. The original gel images of Fig. 5E. Fig. S8. Combination of olaparib-Ga with cisplatin or carboplatin activates ATM/ATR-Chk1/Chk2 pathways in SKOV3-cis and A2780-cis cells, related to Fig. 5. Fig. S9. Olaparib-Ga in combination with cisplatin or carboplatin blocks cell cycle progression at S and G2/M phases in SKOV3-cis and A2780-cis cells, related to Fig. 6. Fig. S10. Co-treatment of olaparib-Ga and cisplatin or carboplatin does not cause noticeable hematologic and histological toxicity in healthy mice, related to Fig. 8. [file research.0070.f1.docx]

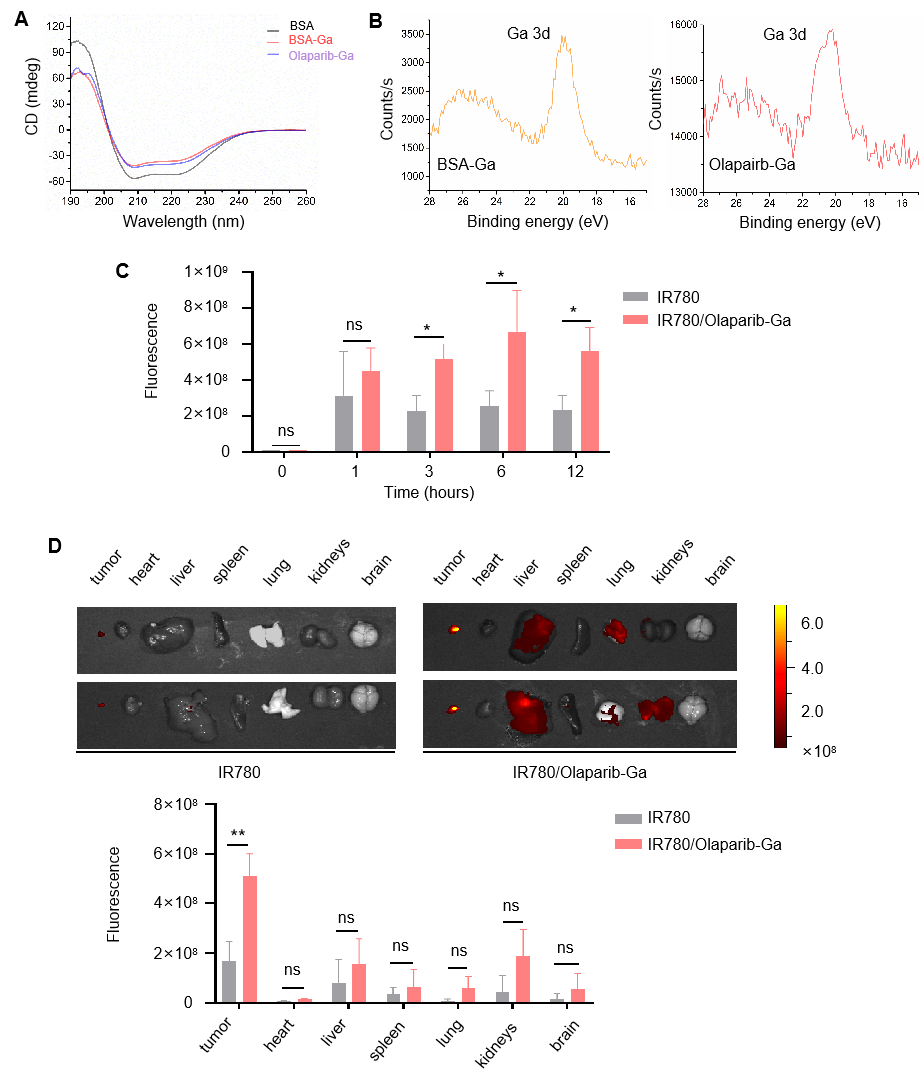


**Fig S1.** Features of olapairb-Ga, relative to figure 1. (A) The CD spectra of pure BSA, BSA-Ga and olaparib-Ga. The CD spectra of BSA-Ga and olaparib-Ga showed no significant variation compared with that of pure BSA protein, proving that the secondary structure of BSA protein did not change during the preparation of olaparib-Ga. (B) The corresponding Ga 3d peak in the XPS survey of BSA-Ga (left) and olaparib-Ga (right). (C) The corresponding quantitative fluorescence analysis of the biodistribution profiles of olaparib-Ga in treated mice was provided, relative to figure 1J. Data are shown as the mean ± SD. (D) The replicated fluorescence images of the xenograft tumor and major organs at 24 h post injection were shown, relative to figure 1K. The corresponding quantitative fluorescence analysis is shown as the mean ± SD. * p < 0.05, ** p < 0.01, *** p < 0.001 vs. the respective control; NS, not significant.


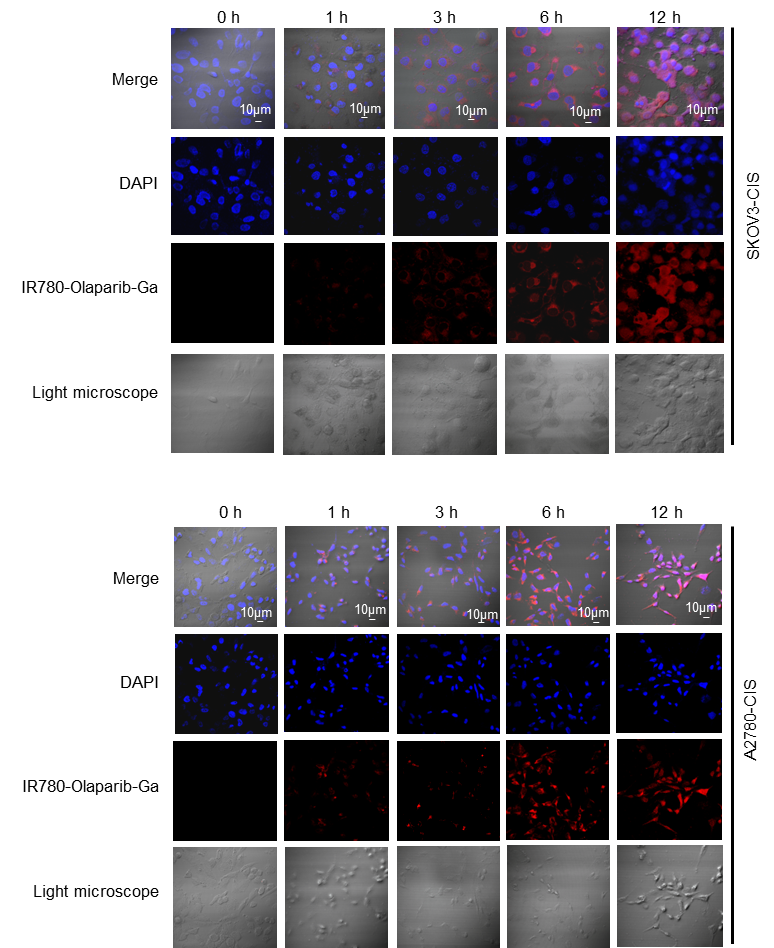


**Fig. S2.** Cellular uptake performance of olaparib-Ga in SKOV3-cis and A2780-cis cells, relative to figure 1. SKOV3-cis and A2780-cis cells were treated with IR780-labeled olaparib-Ga (IR780/olaparib-Ga). Representative images was captured at indicated time points by a confocal laser scanning microscope for 0 h, 1 h, 3 h, 6 h, or 12 h. The nuclei was stained with DAPI (blue). Scale bar, 10 μm.


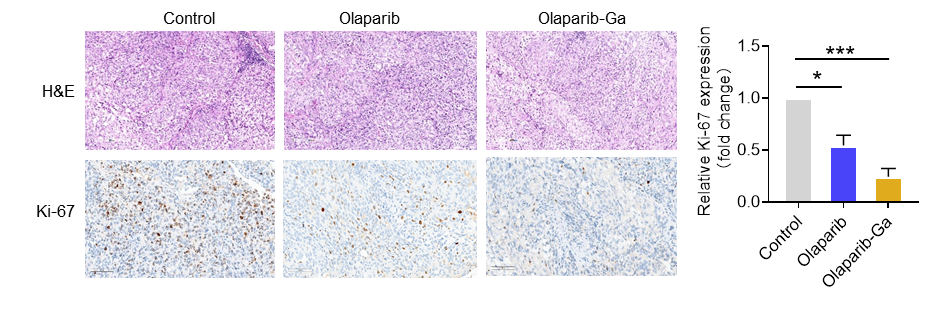


**Fig. S3.** Olaparib-Ga inhibits ki-67 expression of SKOV3-cis-derived xenograft tumors, related to figure 3. H&E and ki-67 immunohistochemistry analysis of xenograft tumors after treatment with olaparib or olaparib-Ga. Scale bar, 200 μm. * p < 0.05, relative to the respective control.


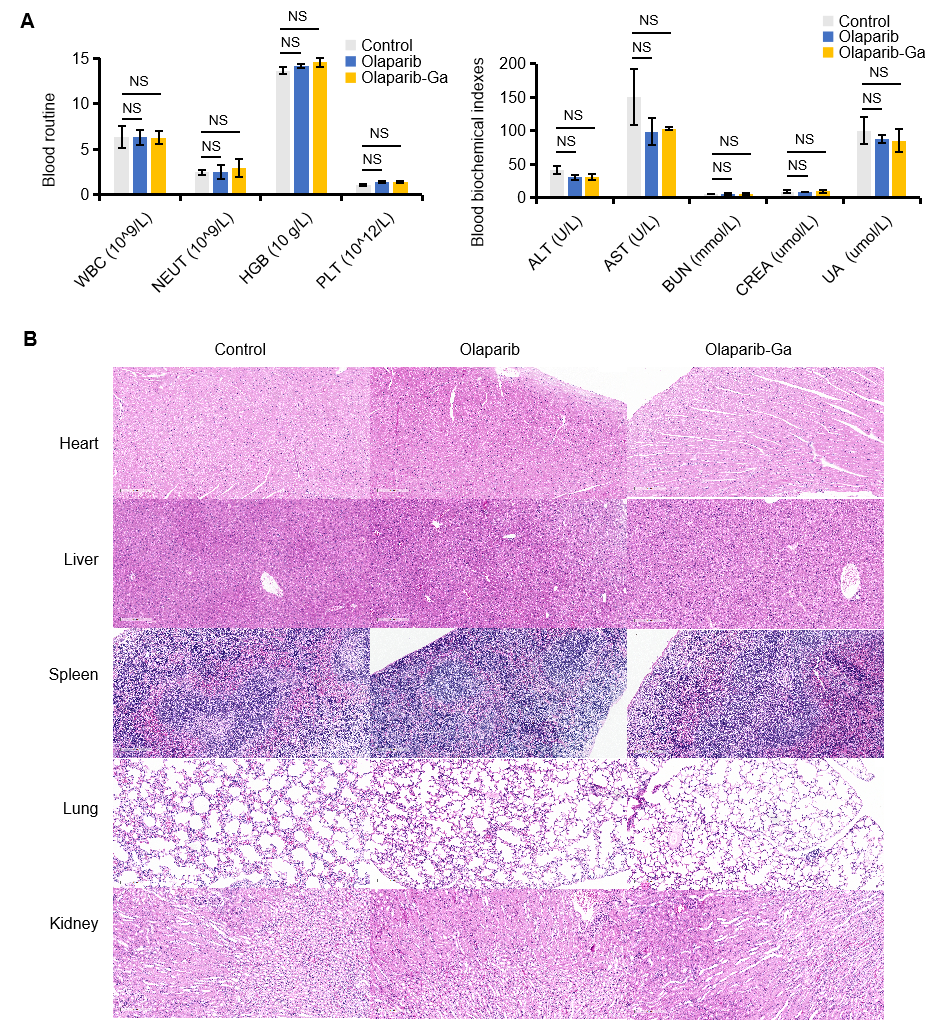


**Fig. S4.** Olaparib-Ga do not induce severe toxicity of SKOV3-cis-derived xenograft mice, related to figure 3. (A) Indicated hematological parameters including blood routine and blood biochemistry were examined in luciferase SKOV3-cis xenograft-bearing mice treated with olaparib or olaparib-Ga . (B) Representative H&E staining images of main organs in different treatment groups are shown. Scale bar, 200 μm. NS, not significant.


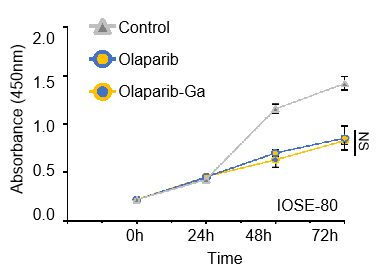


**Fig. S5.** Olaparib-Ga do not induce severe toxicity in human normal ovarian cell lines, related to figure 3. The cell viability curves of olaparib-Ga showing no significant effect on cell viability of IOSE-80 cells, when compared with olaparib, as detected by CCK8 assay. NS, not significant.


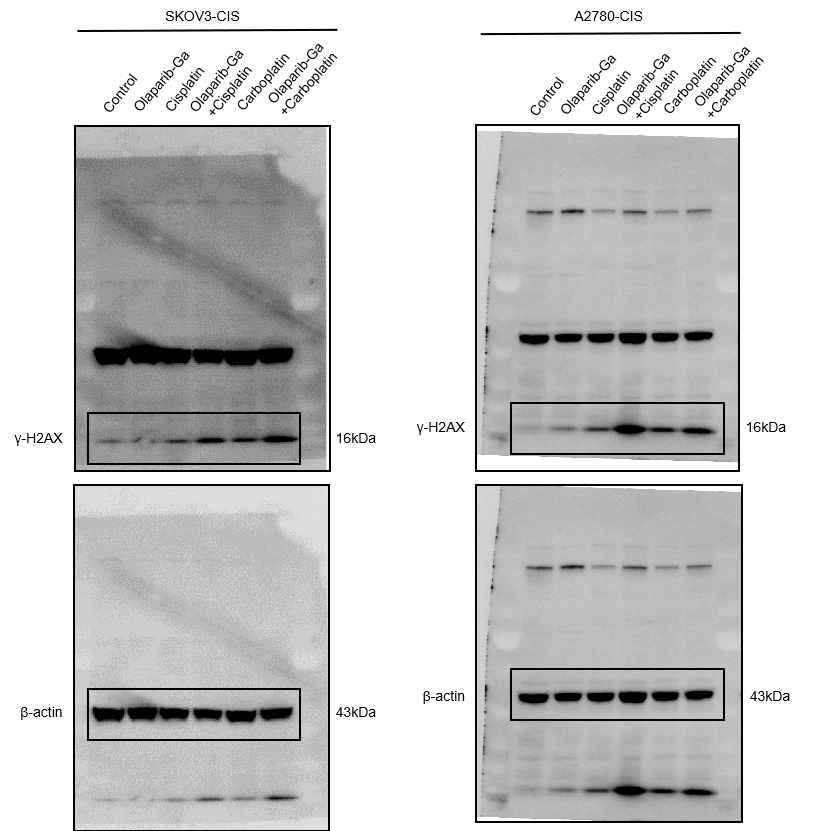


**Fig. S6.** The original gel images of Figure 5B.


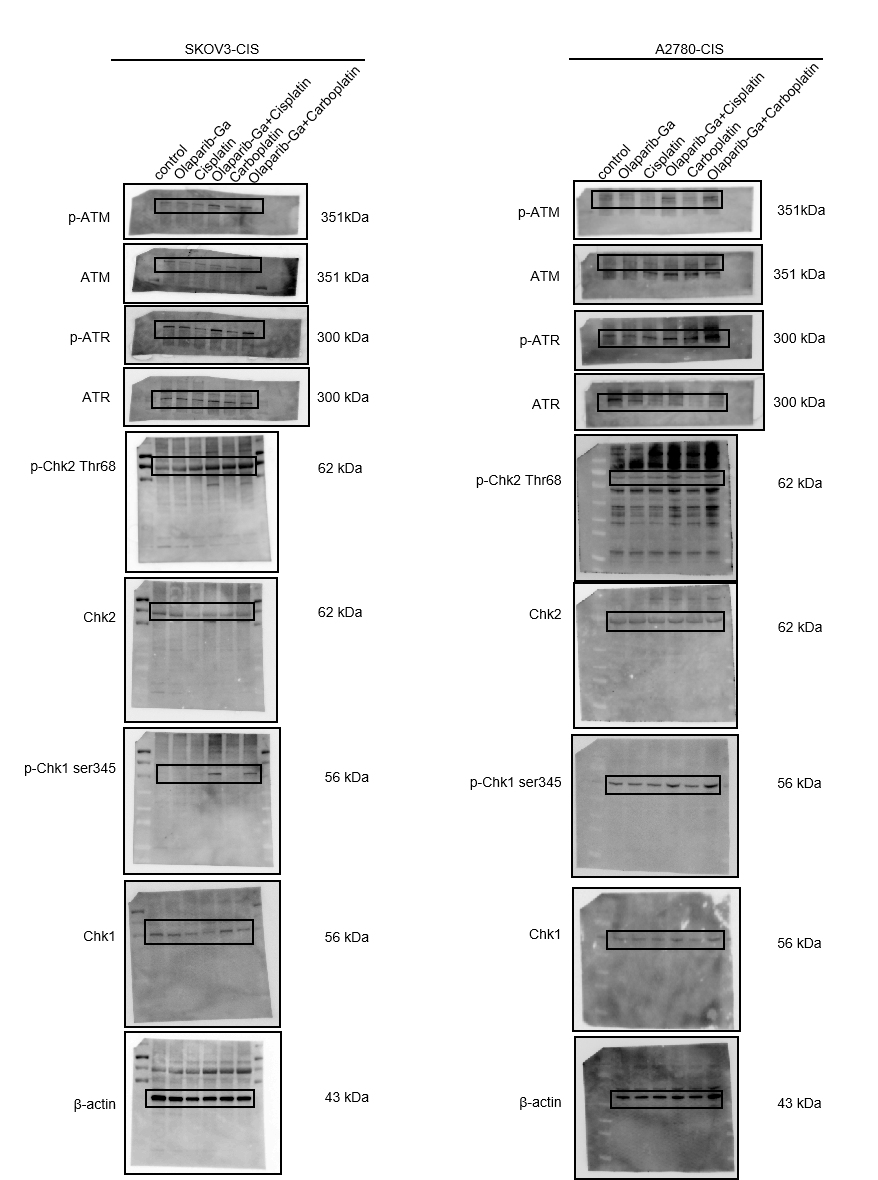


**Fig. S7.** The original gel images of Figure 5E.


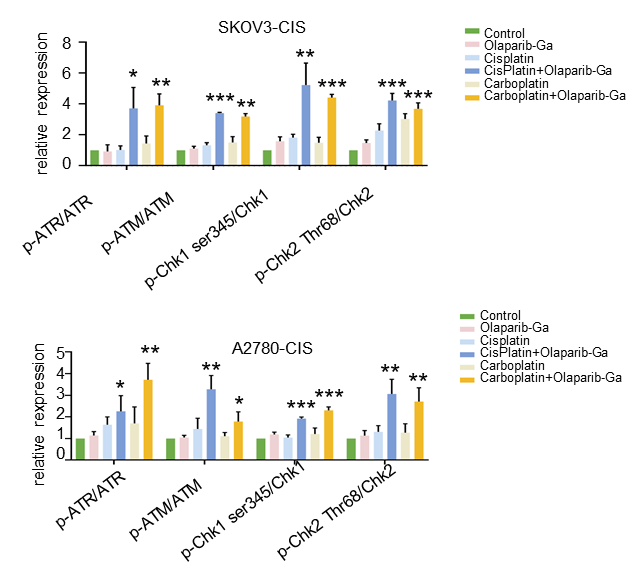


**Fig. S8.** Combination of olaparib-Ga with cisplatin or carboplatin activates ATM/ATR-Chk1/Chk2 pathways in SKOV3-cis and A2780-cis cells, related to figure 5. Bar graphs represent the relative expression ratios of p-ATM vs. ATM, p-ATR vs. ATR, p-Chk1 vs. Chk1, and p-Chk2 vs. Chk2 ± SD. of experimental triplicates. * p < 0.05, ** p < 0.01, *** p < 0.001, relative to the respective control.


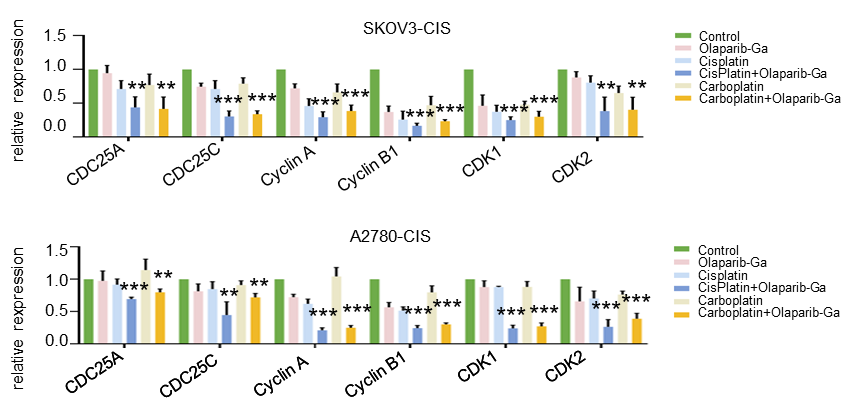


**Fig. S9.** Olaparib-Ga in combination with cisplatin or carboplatin blocks cell cycle progression at S and G2/M phases in SKOV3-cis and A2780-cis cells. , related to figure 6. Bar graphs represent the relative protein expression levels of CDC25A, CDC25C, cyclin A, cyclin B1, CDK1, and CDK2 ± SD. of experimental triplicates. * p < 0.05, ** p < 0.01, *** p < 0.001, relative to the respective control.


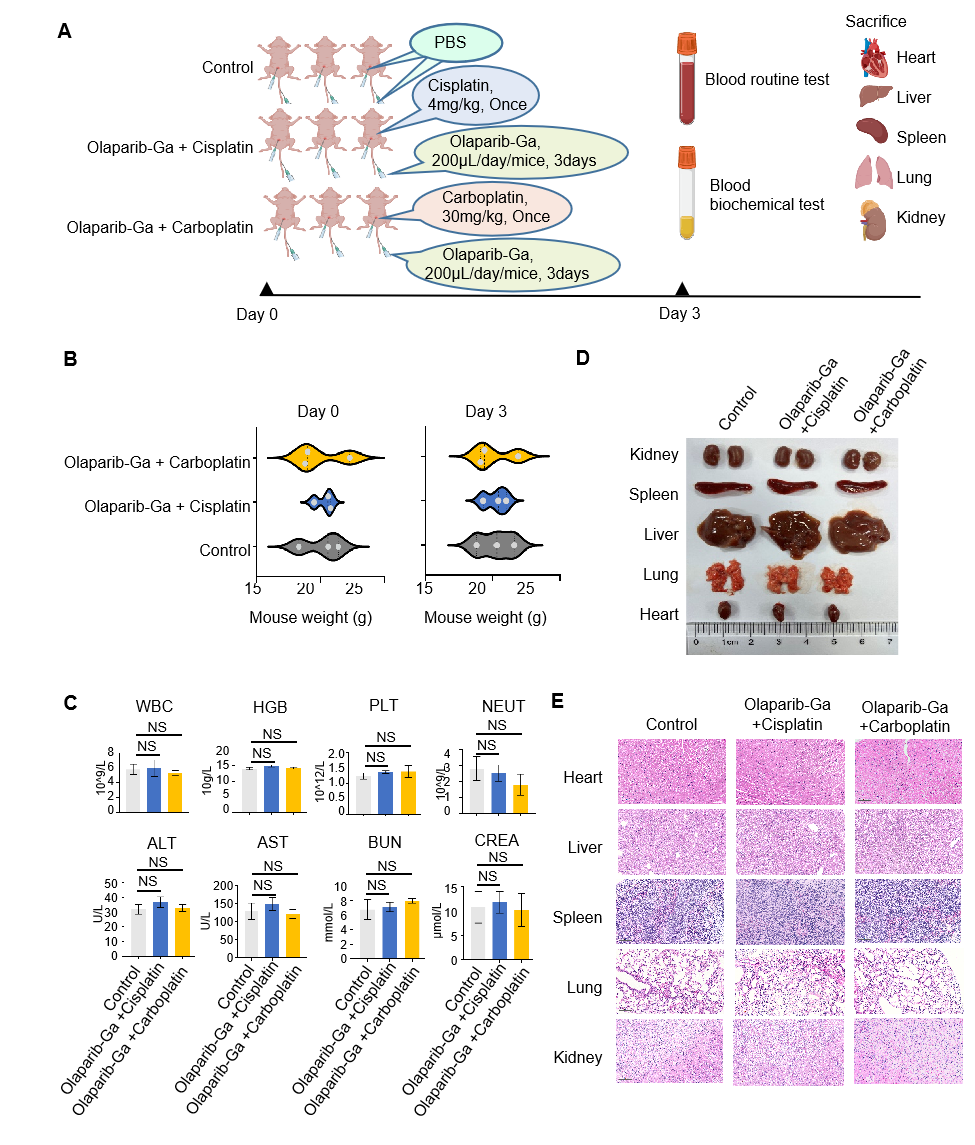


**Fig. S10.** Co-treatment of olaparib-Ga and cisplatin or carboplatin do not cause noticeable hematologic and histological toxicity in healthy mice, related to figure 8. (A) Heathy mice were given intravenous injections of either PBS, co-treatment of olaparib-Ga and cisplatin, or co-treatment of olaparib-Ga and carboplatin at indicated dose per mouse (n=3 in each group). (B) Mouse body weight was tested at indicated time. (C) After 24 h post the last injections, major organs were blood samples were collected for hematology examination. The results of blood routine and blood biochemistry tests were in the normal range. (D and E) Major organs were obtained, imaged (D), and subjected to H&E staining (E). No observable toxicity was found in the tissues from the organs. Scale bar, 200 μm. NS, not significant.
